# Navigational Style Influences Eye Movement Pattern during Exploration and Learning of an Environmental Map

**DOI:** 10.3389/fnbeh.2016.00140

**Published:** 2016-06-29

**Authors:** Laura Piccardi, Maria De Luca, Raffaella Nori, Liana Palermo, Fabiana Iachini, Cecilia Guariglia

**Affiliations:** ^1^Department of Life, Health and Environmental Science, University of L’AquilaL’Aquila, Italy; ^2^Neuropsychology Unit, IRCCS Fondazione Santa LuciaRome, Italy; ^3^Department of Psychology, University of BolognaBologna, Italy; ^4^Department of Medical and Surgical Science, University Magna GraeciaCatanzaro, Italy; ^5^Department of Psychology, “Sapienza” University of RomeRome, Italy

**Keywords:** human navigation, map learning, spatial cognitive style, landmark, route, survey, eye-movements

## Abstract

During navigation people may adopt three different spatial styles (i.e., Landmark, Route, and Survey). Landmark style (LS) people are able to recall familiar landmarks but cannot combine them with directional information; Route style (RS) people connect landmarks to each other using egocentric information about direction; Survey style (SS) people use a map-like representation of the environment. SS individuals generally navigate better than LS and RS people. Fifty-one college students (20 LS; 17 RS, and 14 SS) took part in the experiment. The spatial cognitive style (SCS) was assessed by means of the SCS test; participants then had to learn a schematic map of a city, and after 5 min had to recall the path depicted on it. During the learning and delayed recall phases, eye-movements were recorded. Our intent was to investigate whether there is a peculiar way to explore an environmental map related to the individual’s spatial style. Results support the presence of differences in the strategy used by the three spatial styles for learning the path and its delayed recall. Specifically, LS individuals produced a greater number of fixations of short duration, while the opposite eye movement pattern characterized SS individuals. Moreover, SS individuals showed a more spread and comprehensive explorative pattern of the map, while LS individuals focused their exploration on the path and related targets. RS individuals showed a pattern of exploration at a level of proficiency between LS and SS individuals. We discuss the clinical and anatomical implications of our data.

## Introduction

How does the brain build up a map of the environment and how can individuals navigate through a complex space? The answer to these questions has been partially provided by the discovery of place (O’Keefe and Dostrovsky, 1971) and grid cells (Hafting et al., 2005), components of a “positioning system” that allows rats to localize a specific area in a space (Moser et al., 2015). However, how human beings acquire and process information is still matter of debate (Nori and Piccardi, 2011). Although, much evidence points to the fact that place cells exist also in humans (Ekstrom et al., 2003) and cognitive maps of the environment are developed and continuously updated to allow an active process of environmental navigation (Maguire et al., 1999), the nature and neural correlates of this system are still a matter of investigation. Importantly, at variance with rodents that mainly gather information on an environment by moving to explore different locations and using their impressive olfactory abilities, primates use eye movements to visually explore an environment, allowing inspection of the environment also at a distance (Schiller et al., 2015). Indeed, recent results showed grid cells in the entorhinal cortex of primates during visual exploration without locomotion, suggesting that spatial representations in primates can arise during visual exploration at a distance (Killian et al., 2012).

According to Siegel and White’s (1975) model, different kinds of environmental knowledge can be acquired and represented depending on the type of information selected: *landmark knowledge*, by which an individual “beacons” toward environmental patterns perceptually salient or important for him/her, is a sort of figurative memory; *route knowledge*, by which an individual navigates relying on the memory of the paths that connect different landmarks, is organized on the basis of body references, that is, an egocentric frame of references; and *survey knowledge*, which corresponds to a global representation of the environment, like a map (i.e., a cognitive map), allows new paths to be found between different locations and implies the encoding of directions and distances between places regardless of the person’s position, that is, allocentric frames of reference or coordinates (e.g., north, south, east, and west [cardinal points]). According to the Authors, these representations are hierarchically organized and develop at different ages. Recently, researchers have disagreed in regards to the hierarchical representation model. Namely, Siegel and White’s model is cumulative because landmark representation is characterized only by its properties, while; route representation is characterized by the features of landmark and route’s representations; and survey representation includes the features of all representations. Montello (1998) hypothesizes that individuals may acquire an overall survey representation right from their very first exposure to an environment and people with equal levels of familiarity with an environment differ in the extent and accuracy of their spatial knowledge. Pazzaglia et al. (2000) suggest that the three phases identified in Siegel and White’s model could correspond to three different spatial cognitive styles (SCSs) that the individual may adopt during navigation. According to this proposal, people’s behavior during navigation could be classified as landmark (LS), route (RS), or survey style (SS). These three different styles also represent three different levels of ability in navigation. That is to say, people with LS are less proficient in navigation and experience more frequently the feeling of getting lost, while people with route style are more able to correctly decide where and when to turn right or left if a specific landmark is present along the path, and people with SS are very proficient navigators able to retain the spatial layout of an environment, find a shortcut between two locations or create an interconnected network among different paths without the aid of specific landmarks (Nori and Piccardi, 2011).

Individual SCSs help to explain why some people are good at finding their way back to a starting position along a path that they only experienced once, whereas others fail to do so (Kozlowski and Bryant, 1977; Sholl et al., 2006).

Nori et al. (2009) also suggested that the person’s SCS is influenced by personality and social factors, and Lawton (1994) previously pointed out the relationship between SCS and spatial anxiety, which is a personality trait that can be defined as the tendency to experience fear of getting lost in the environment.

Much evidence points to the fact that differences in navigation ability are related to several internal (personal) and external (environmental) factors, such as sex (with men outperforming women; Montello et al., 1999; Halpern, 2000; Palermo et al., 2008; Piccardi et al., 2008, 2015; Nori and Piccardi, 2015; Nori et al., 2015b), characteristics of the spatial tasks (Freundschuh et al., 1990; Prestopnik and Roskos-Ewoldsen, 2000; Piccardi et al., 2011a,b, 2014; Nori et al., 2015a), and complexity of the spatial layout; in this latter case, simpler layouts facilitate the maintenance of direction, and increase the chance of choosing the right route to reach a given goal (Evans et al., 1984; Gärling et al., 1986).

The studies described above show that the SCS influences the proficiency in orienting through an environment, perhaps by affecting the way individuals explore environments, pay attention to different types of environmental features (for example, paying more attention to the geometrical features rather than to the color of the landmarks), and select the information to be coded and stored. These different strategies used by people to move through the environment could also help us to better understand navigational learning disabilities such as the developmental topographical disorientation (DTD; Iaria et al., 2009; Bianchini et al., 2010, 2014; Palermo et al., 2014b,a; Piccardi et al., under review), a deficit that could be widespread among the population (see Iaria and Barton, 2010). As healthy individuals with different cognitive spatial styles could show different *normal* patterns of eye movements during the exploration of an environment, individuals with different types of DTD could be characterized by different *pathological* patterns of eye movements. The aim of the present study is to investigate whether a particular SCS, determined through standardized tests, affects the visual exploration of a novel environment. We hypothesize that the individuals’ spatial style may determine the way in which people observe, and therefore acquire, information from an environment. To this purpose we classified participants according to their SCS (i.e., LS; RS; and SS), and recorded their eye movements during the learning and delayed recall of a path depicted on a simplified map in which the cardinal points were shown. Our hypothesis is that the way people process spatial information is reflected by their eye movement pattern during spatial tasks (that is, people sharing the same navigational style also share common eye movement patterns during the exploration of a map), and is related to how successful they could be in orienting themselves through the environment.

## Materials and Methods

### Participants

Fifty-one healthy right-handed College Students (mean age 25.1 ± 3.4 years; 30 women), without neurological or psychiatric disorders, participated in the study, which was approved by the local ethics committee of the Department of Psychology at the “Sapienza” University of Rome, in agreement with the Declaration of Helsinki; all participants gave their written informed consent.

On the basis of scores obtained at the SCS test (Nori and Giusberti, 2006; see Tasks and stimuli for description), participants were classified into three groups. Specifically, 20 participants (14 females) were included in the LS group (SCST total score: 14.7 ± 1.7); 17 (12 females) in the Route Style group (RS; SCST score: 16.0 ± 0.4; 12 women), and 14 (four females) in the SS group (SCST score: 17.6 ± 0.6; four women).

### Tasks and Stimuli

The general sense of direction was evaluated in all participants by the Familiarity and Spatial Cognitive Style scale (FSCS; Piccardi et al., 2011a) which includes 22 self-referential statements about various aspects of environmental spatial cognition. Participants responded by rating items on a five-point scale with anchors (1) Very poor and (5) Excellent. The FSCS was used to exclude participants with self-declared topographical orientation disorders. None of the participants showed the presence of navigational deficits or DTD (see Iaria et al., 2005, 2009; Bianchini et al., 2010).

**FIGURE 1:**
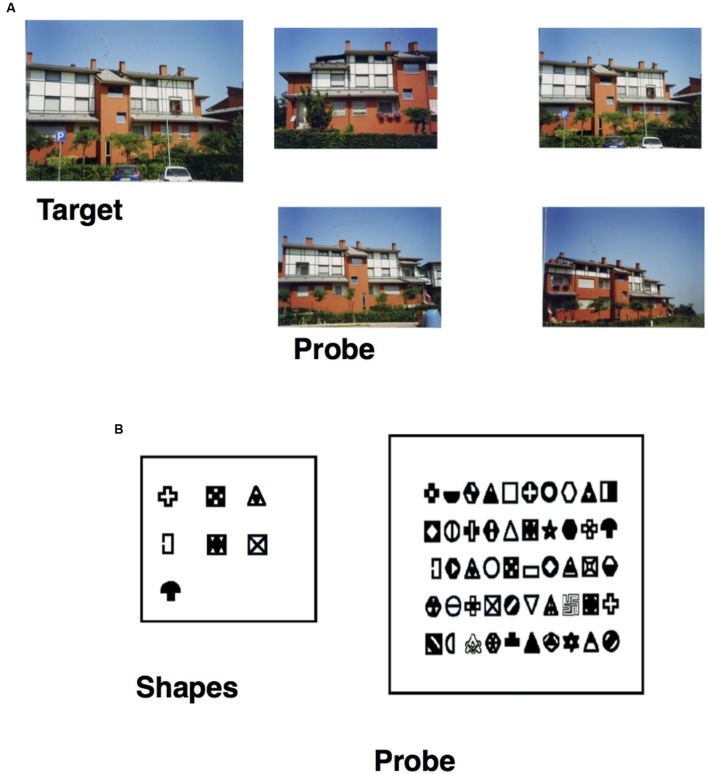
**(A)** Item of the Photo task, specifically the target building (on the left) and the probe (on the right), in which the participant has to select the target previously studied. **(B)** Item of the Figure task, specifically the seven shapes (on the right) the participant has to study and the probe (on the left) in which he/she has to recognize them from among 50 shapes.

**FIGURE 2:**
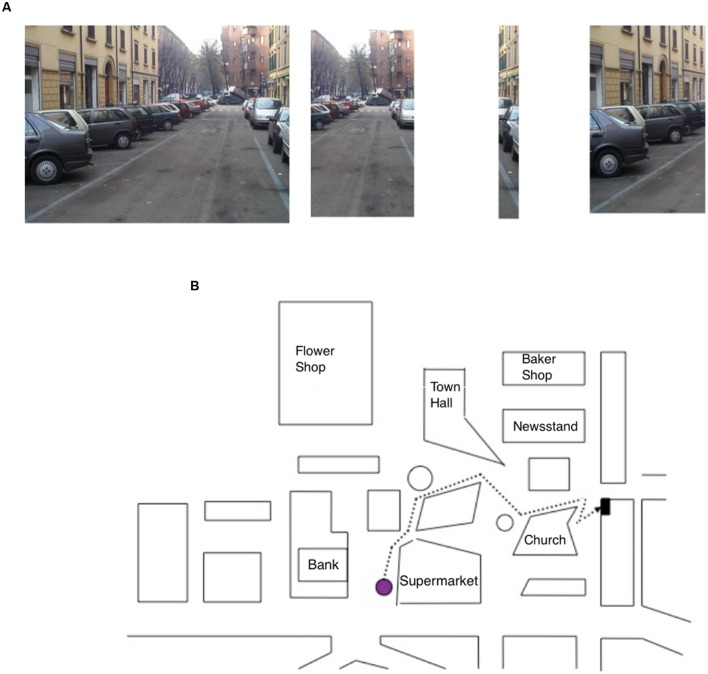
**(A)** Item of the Sequence task, specifically the photo target (on the right) depicting a navigational scene and the parts (on the left) into which the photo has been subdivided; the task of the participant was to put the parts into the correct order. **(B)** Map description task, in which the participant has to describe the route (starting from the purple dot) to reach the goal while reporting the correct sequence of seven right–left turning points.

**FIGURE 3:**
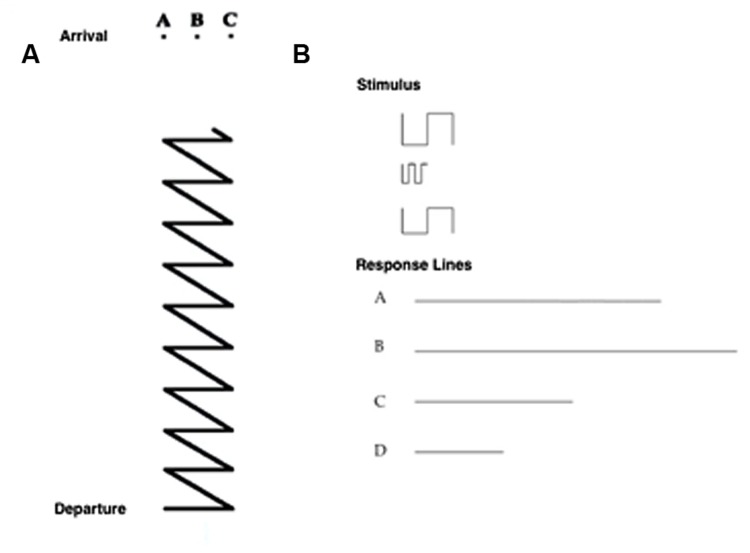
**(A)** Item of the Path Task, in which the participant has to choose the upper ending point of a path from among three possibilities. **(B)** Item of the Sum and Straighten task, in which the participant has to mentally sum and straighten a series of segments (above) to obtain the actual length, and then to indicate the correct answer from among four alternatives (a, b, c, d; below).

The SCS test (Nori and Giusberti, 2006) was used to classify the participants according to the three navigational styles (LS; RS; SS). The test, in its short version, included the following six subtests:

• *Photo task*: participants were asked to select a building-target (previously studied for 3 s) from among four photos of similar buildings (seven trials; see **Figure 1A**).

• *Figure task*: participants had to study seven shapes for 75 s and were then asked to recognize them from among 50 shapes (seven targets and 43 fillers; see **Figure 1B**).
• *Sequence task*: participants were asked to study for 15 s, a photo representing a navigational scene from a first-person perspective. The navigational scene was then divided into separate parts (3, 4, or 5) and the participants’ task was to arrange them in the correct order so as to reconstruct the previously studied photo (seven trials; **Figure 2A**).

• *Map Description task*: participants were asked to describe a pathway depicted on a map; starting from a purple dot, they had to describe the route to reach a black dot (representing the navigation goal) by reporting the correct sequence of seven right–left turning points. Rotation of the map was explicitly required to perform the task (see **Figure 2B**).
• *Path task:* participants had to choose the upper ending point of a path from among three possibilities (seven trials; **Figure 3A**).

• *Sum and Straighten task:* participants had to mentally sum and straighten a series of segments on a piece of paper to obtain the actual length, and then indicate the correct answer from among four alternatives (seven trials; **Figure 3B**).

On the basis of the cumulative model’s characteristics as above described and following the criteria of Nori and Giusberti (2003, 2006), we classified participants as Landmark cognitive style if they provided at least 80% of correct answers on the landmark tasks and those who provided 50% (chance level) or fewer correct answers on both the route and survey tasks. Participants who gave at least 80% of correct answers in both the landmark and route tasks and 50% (chance level) or fewer correct answers in the survey tasks, were classified as Route cognitive style and finally, participants who provided at least 80% of correct answers in the landmark, route and survey tasks were considered as Survey cognitive style.

#### Navigational Task

**FIGURE 4:**
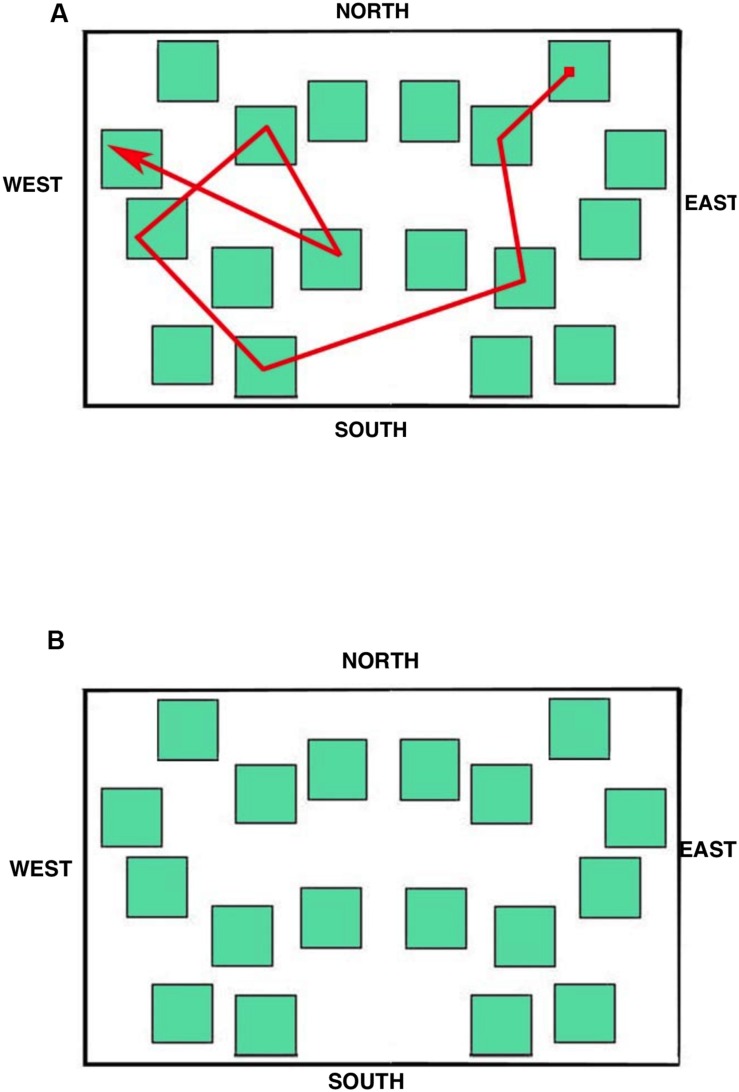
**Maps used during the learning **(A)** and the delayed recall **(B)** phases**.

The stimulus displayed during the eye movement recording was a simplified city map (subtending 15.5° × 10.0° of visual angle at the viewing distance of 57 cm; **Figure 4A**) provided with the four cardinal points in order to allow participants to orient themselves during the task. The map was made up of 18 green square blocks each subtending 1.5° × 1.5°, displayed on a white background. A pathway, that connected eight squares (target blocks) and included one intersection, was depicted with a red line; starting and ending points were indicated by a small filled red square and by an arrow, respectively.

#### Learning Phase

Participants were required to study the map silently for 220 s (i.e., the maximum time to learn the path, as estimated in Piccardi et al. 2011b). Participants were also asked to click the mouse when they believed they had learned the pathway, but were instructed to continue studying the map until it disappeared from the screen even after they had pressed the mouse. The subjective time of learning (time elapsing from the map presentation and the mouse click) was also recorded. Immediately at the end of the learning phase there was a 5-min interval during which participants answered an anamnesis questionnaire including questions about the participant’s age, gender, addictions, and general state of health.

#### Delayed Recall Phase

Immediately after the interval, the participants were presented with the same map on the screen without any indication of the pathway (see **Figure 4B**). They were required to indicate the blocks included in the pathway in the correct order from the starting point to the ending point by clicking the mouse within each block. The participants were also told that if they realized they had made a mistake it was not possible to correct it or to start again from the beginning, but simply to complete the sequence from the following block on. No feedback was provided and there was no time limit. The number of correctly selected target blocks (range: 0–8) and the achievement of the correct whole sequence were scored.

### Eye Movement Recordings

The eye movements were recorded during the learning and the delayed recall phases.

#### Apparatus and General Procedure

Eye movements from the dominant eye were recorded in binocular vision via an SR Research, Ltd. Eye Link 1000 eye tracker (SR Research, Ltd., Mississauga, ON, Canada) sampling at 1000 Hz, with spatial resolution of less than 0.04°. Head movements were avoided by using a headrest and a chinrest. Stimuli were displayed on a 17″ CRT monitor at a viewing distance of approximately 57 cm. A nine-point calibration procedure was run before the beginning of each phase. The calibration targets were presented randomly in different positions on the screen. The appearance of the map on the screen was automatically triggered by a steady (for at least 250 ms) eye fixation of a cross immediately after calibration. The recording script was programmed to allow using the mouse as a manual response device to select the blocks: mouse click events were time-stamped and mouse positions were continuously and digitally recorded and stored along with eye movements; in the delayed recall phase, this was needed to measure the accuracy and timing of the recalled pathway. The default arrow-shape of the mouse cursor was changed into a white equilateral triangle with black edges.

#### Data Analysis

Eye movement data were processed through EyeLink Data Viewer software (SR Research, Ltd., Mississauga, ON, Canada). Fixations landing on the eight target blocks, on the 10 non-target blocks, and on the four cardinal points were considered separately; also the fixations landing on the white area of the map enclosing the blocks were included. For each participant, separately for the two phases, the following eye movement parameters were measured: (a) the total number of fixations made to perform either task, and the percentages of fixations (on the total) separately for fixations landing on targets, non-targets, cardinal points, and white area; (b) the average fixation duration, and the mean fixation duration separately for targets, non-targets, cardinal points and white area; (c) the total dwell time (i.e., the sum of all fixation durations), and the percentage of dwell time separately for targets, non-targets, cardinal points, and white area; (d) the total number of runs within all elements (i.e., a run – or first pass – is made by consecutive fixations within the same interest-area before moving to another interest-area), and the percentage of runs separately for targets, non-targets, and cardinal points; finally, (e) the mean saccade amplitude. Separate one-way ANOVAs with Group (LS, RS, SS) as between factor were carried out on all eye movement parameters. *Post hoc* tests (Duncan) were run in case of significant comparisons.

Various behavioral data were measured for each participant. In the *Learning phase* the subjective time of learning (i.e., the time at which the participant clicked the mouse because he/she was confident to have learned the pathway) was recorded, and the number of fixated target (maximum 8) and non-target (maximum 10) blocks was computed. In the *Recall phase* the total time of execution (i.e., from stimulus onset until the subject selected the last block), the time elapsing from the first until the last block selection, the number of fixated target (maximum 8) and non-target blocks (maximum 10), the number of selected targets (i.e., the correct blocks regardless of sequence), the number of selected non-targets. Separate one-way ANOVAs with Group (LS, RS, SS) as between factor were carried out on behavioral measures. Finally, the accuracy of the sequence (whether or not the eight selected targets were clicked in the correct sequential order) was determined and the percentage of participants of each group that recalled the pathway without making any mistake was computed. The alpha level was set at 0.05.

## Results

Behavioral and eye movement results are reported in **Table 1**.

**Table 1:** Socio-demographic data.

	Landmark (six males; 14 females)		Route (five males; 12 females)		Survey (10 males; four females)		*p*-value
				
	Mean	*SD*	Mean	*SD*	Mean	*SD*	
Age (years)	24.8	*3.6*	25.3	*3.2*	25.2	*3.6*	
Education (years)	15.5	*1.8*	15.9	*2.1*	15.1	*3.0*	
SCST^1^	14.7	*1.7*	16.0	*0.4*	17.6	*0.6*	
**Learning phase**							
Subjective time of learning (s)	51.2	*37.5*	63.0	*46.9*	46.8	*25.3*	n.s.
Number of fixated target blocks (maximum 8)	7.95	*0.2*	8.00	*0.0*	7.93	*0.3*	n.s.
Number of fixated non-target blocks (maximum 10)	8.10	*1.4*	7.41	*2.0*	7.57	*1.1*	n.s.
Total number of fixations	549	*104*	458	*82*	474	*106*	0.016
Percentage of fixations for targets	59.3	*9.9*	62.6	*9.9*	52.7	*10.1*	0.028
Percentage of fixations for non-targets	8.7	*3.2*	7.7	*3.7*	11.3	*3.3*	0.015
Percentage of fixations for cardinal points	2.8	*2.1*	2.6	*1.6*	1.5	*1.2*	n.s. (0.09)
Percentage of fixations for white area	29.3	*7.7*	27.1	*9.1*	34.5	*8.4*	0.054
Fixation duration (ms)	315	*51*	360	*55*	375	*63*	0.007
Fixation duration for targets (ms)	335	*49*	391	*63*	389	*60*	0.006
Fixation duration for non-targets (ms)	286	*63*	291	*53*	359	*100*	0.012
Fixation duration for cardinal points (ms)	251	*64*	236	*51*	303	*112*	n.s. (0.06)
Fixation duration for white area (ms)	280	*62*	308	*53*	358	*86*	0.007
Total dwell time (s)	169.1	*21.6*	163.1	*26.6*	172.5	*23.4*	n.s.
Percentage of dwell time for targets (s)	63.3	*11.4*	67.9	*11.1*	55.0	*11.0*	0.009
Percentage of dwell time for non-targets (s)	7.9	*3.1*	6.4	*3.3*	10.8	*3.5*	0.002
Percentage of dwell time for cardinal points (s)	2.4	*2.1*	1.9	*1.3*	1.2	*1.0*	n.s. (0.15)
Percentage of dwell time for white area (s)	26.4	*8.9*	23.8	*10.2*	33.0	*9.8*	0.032
Total number of runs	309.5	*60.5*	269.9	*44.8*	260.2	*73.9*	0.043
Percentage of runs for targets (s)	78.4	*7.0*	81.1	*6.4*	76.6	*8.1*	n.s.
Percentage of runs for non-targets (s)	16.8	*5.9*	14.5	*6.1*	20.7	*7.5*	0.034
Percentage of runs for cardinal points (s)	4.8	*2.9*	4.4	*2.9*	2.7	*2.1*	n.s. (0.08)
Saccade amplitude (deg)	2.53	*0.51*	2.60	*0.49*	2.66	*0.51*	n.s.
**Recalling phase**							
Total time of execution (s)	62.9	*53.5*	62.2	*65.8*	45.0	*40.0*	n.s.
Time from first to last block selection (s)	36.8	*31.9*	33.8	*37.6*	18.5	*10.8*	n.s.
Number of fixated target blocks (maximum 8)	7.5	*0.8*	*7.6*	0.6	7.2	*1.1*	n.s.
Number of fixated non-target blocks (maximum 10)	5.5	*2.4*	4.4	*2.6*	4.1	*2.7*	n.s. (0.075)
Number of selected targets (i.e., correct responses, maximum 8)	6.2	*0.8*	6.9	*1.2*	7.6	*0.9*	0.001
Number of selected non-targets (i.e., wrong responses, maximum 10)	1.4	*0.9*	0.9	*0.9*	0.4	*0.9*	0.016
Total number of fixations	144	*100*	134	*149*	103	*102*	n.s.
Percentage of fixations for targets	47.8	*7.0*	55.6	*9.8*	51.9	*15.7*	n.s.
Percentage of fixations for non-targets	23.7	*6.8*	19.3	*9.2*	14.6	*6.4*	0.005
Percentage of fixations for cardinal points	1.0	*1.3*	0.8	*1.2*	1.3	*2.1*	n.s.
Percentage of fixations for white area	27.4	*8.7*	24.4	*11.1*	32.2	*15.7*	n.s.
Fixation duration (ms)	321	*60*	377	*74*	410	*123*	0.014
Fixation duration for targets (ms)	354	*70*	411	*81*	465	*172*	0.020
Fixation duration for non-targets (ms)	301	*55*	327	*88*	320	*67*	n.s.
Fixation duration for cardinal points (ms)	223	*65*	272	*63*	191	*62*	0.078
Fixation duration for white area (ms)	278	*67*	309	*92*	351	*92*	0.058
Total dwell time (s)	44.9	*33.6*	47.7	*48.3*	36.0	*28.1*	n.s.
Percentage of dwell time for targets (s)	52.6	*7.7*	60.9	*12.2*	57.5	*16.2*	n.s.
Percentage of dwell time for non-targets (s)	22.8	*7.2*	17.3	*9.6*	12.5	*7.1*	0.002
Percentage of dwell time for cardinal points (s)	0.7	*0.9*	0.5	*0.8*	0.7	*1.1*	n.s.
Percentage of dwell time for white area (s)	23.9	*8.9*	21.3	*12.2*	29.4	*14.4*	n.s.
Total number of runs	93.4	*67.4*	83.5	*85.3*	65.4	*77.0*	n.s.
Percentage of runs for targets (s)	65.4	*7.1*	73.8	*11.4*	75.0	*8.1*	0.005
Percentage of runs for non-targets (s)	32.5	*7.9*	25.1	*10.9*	22.3	*8.2*	0.005
Percentage of runs for cardinal points (s)	2.1	*2.4*	1.1	*1.6*	2.7	*4.7*	n.s.
Saccade amplitude (deg)	2.22	*0.31*	2.27	*0.61*	2.42	*0.50*	n.s.
			
	***N***		***N***		***N***		
			
Number (and percentage) of participants recalling correctly	1 (5%)		7 (41.2%)		9 (64.3%)		


*Assessment scores and eye movements results (mean and standard deviation) separately for the three groups of participants. Statistical level of significance of group comparisons is reported in the last column.*

*^1^SCST, The Spatial Cognitive Style Test (Nori and Giusberti, 2006).*

### Learning Phase

**FIGURE 5:**
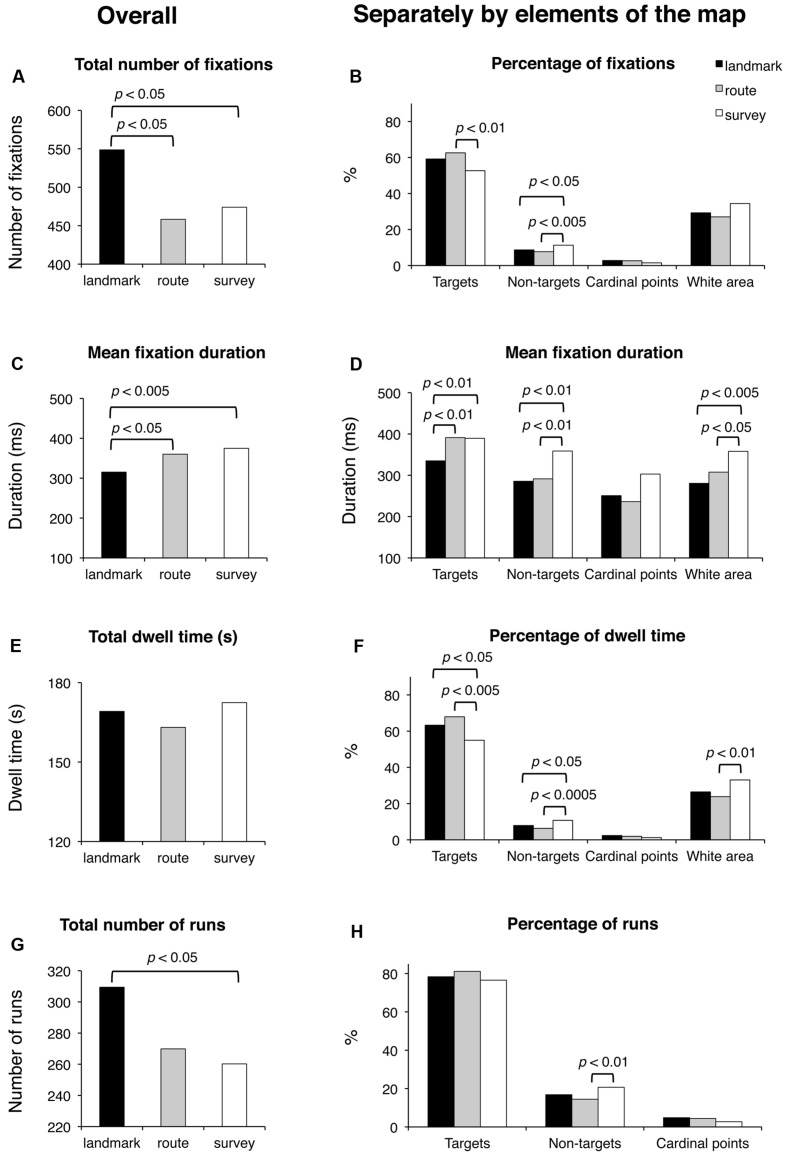
**Learning phase.** Main results are shown for overall (left panels) and for distinct elements of the map (target and non-target blocks, cardinal points, and white area; right panels) for the following eye movement parameters: total number of fixations **(A)**, percentage of fixations **(B)**, mean fixation duration **(C,D)**, total dwell time **(E)**, percentage of dwell time **(F)**, total number of runs **(G)**, and percentage of runs **(H)**.

The results are shown in **Figure 5**. Subjective time of learning and the number of fixated target and non-target blocks were comparable among groups (for all cases, *F*_(2,48)_ < 1; *p* = 0.47, *p* = 0.58, and *p* = 0.39, respectively). Groups differed significantly for the total number of fixations (*F*_(2,48)_ = 4.53; *p* < 0.05). *Post hoc* analysis showed that LS made a greater number of fixations (549) than RS (458; *p* < 0.05) and SS (474; *p* < 0.05), whereas the latter two groups did not differ (*p* = 0.64). A comparison of the percentages of fixations showed that while for cardinal points there were no differences among groups (*F*_(2,48)_ = 2.49; *p* = 0.094), group difference approached significance for the percentage of fixations in the white area (*F*_(2,48)_ = 3.09; *p* = 0.055), and there were significant group differences for the percentage of fixations on targets (*F*_(2,48)_ = 3.86; *p* < 0.05) and non-targets (*F*_(2,48)_ = 4.59; *p* < 0.05); *post hoc* showed that SS (52.7) had a lower percentage of fixations on targets than RS (62.6; *p* < 0.01) and tended to differ from LS (59.2; *p* = 0.064), while the latter two groups did not differ (*p* = 0.34); the percentage of fixations made on non-targets was significantly greater for SS (11.3) with respect to LS and RS (8.7 and 7.7; *p* < 0.05 and *p* < 0.01, respectively), while the latter two groups did not differ (*p* = 0.40). Mean fixation duration (*F*_(2,48)_ = 5.45; *p* < 0.01), mean fixation duration for targets (*F*_(2,48)_ = 5.73; *p* < 0.01), non-targets (*F*_(2_,_48)_ = 4.87; *p* < 0.05), and white area (*F*_(2,48)_ = 5.56; *p* < 0.01) differed significantly among groups. *Post hoc* analyses showed that average duration and over-target duration were significantly shorter in LS (315 and 335 ms, respectively) than in RS (360 and 391 ms; *p* < 0.05 and *p* < 0.01, respectively) and SS (375 and 389 ms, respectively; both *p*s < 0.01), while the latter two groups were comparable (*p* = 0.45 and *p* = 0.93, respectively); instead, the duration of fixations on non-target blocks and white area was significantly longer in SS (359 and 358 ms, respectively) than in RS (291 and 308 ms; *p* < 0.01 and *p* < 0.05, respectively) and LS (286 and 281 ms; *p* < 0.01 and *p* < 0.005, respectively), while the latter two groups were comparable (*p* = 0.82 and *p* = 0.24, respectively). Group difference approached significance for the durations of fixations on cardinal points (*F*_(2_,_48)_ = 3.05; *p* = 0.057). Total dwell time did not differ among groups (*F*_(2,48)_ < 1; *p* = 0.54). Groups significantly differed in the percentage of dwell time for targets (*F*_(2,48)_ = 5.21; *p* < 0.01), non-targets (*F*_(2,48)_ = 7.12; *p* < 0.005), and white area (*F*_(2,48)_ = 3.70; *p* < 0.05). *Post hoc* analyses showed that SS had a lower dwell time percentage for targets (55.0) compared to LS (63.3; *p* < 0.05) and RS (67.9; *p* < 0.005), a greater dwell time percentage for non-targets (10.8) than LS (7.9; *p* < 0.05) and RS (6.3; *p* < 0.0005), and a greater percentage for white area (33.0) compared to LS (26.4; *p* < 0.05) and RS (23.8; *p* < 0.05); LS and RS were comparable for the three above-mentioned elements of the map (*p* = 0.24, *p* = 0.17, and *p* = 0.43, for targets, non-targets, and white area, respectively). The groups differed significantly for the total number of runs (*F*_(2,48)_ = 3.36; *p* < 0.05); *post hoc* analysis showed that LS made a greater number of runs (309) compared to SS (260; *p* < 0.05) and tended to differ significantly from RS (270; *p* = 0.063); SS and RS did not differ (*p* = 0.64). The percentage of runs toward targets was comparable among the three groups (*F*_(2,48)_ = 1.65; *p* = 0.20). The percentage of runs toward non-targets showed a group difference (*F*_(2,48)_ = 3.64; *p* < 0.05); *post hoc* showed that SS (20.7) differed significantly from RS (14.5; *p* < 0.01), and almost significantly from LS (16.8; *p* = 0.89); LS and RS did not differ (*p* = 0.30). Finally, saccade amplitude (*F*_(2_,_48)_ < 1; *p* = 0.78) showed no significant difference among the groups.

#### Delayed Recall Phase

**FIGURE 6:**
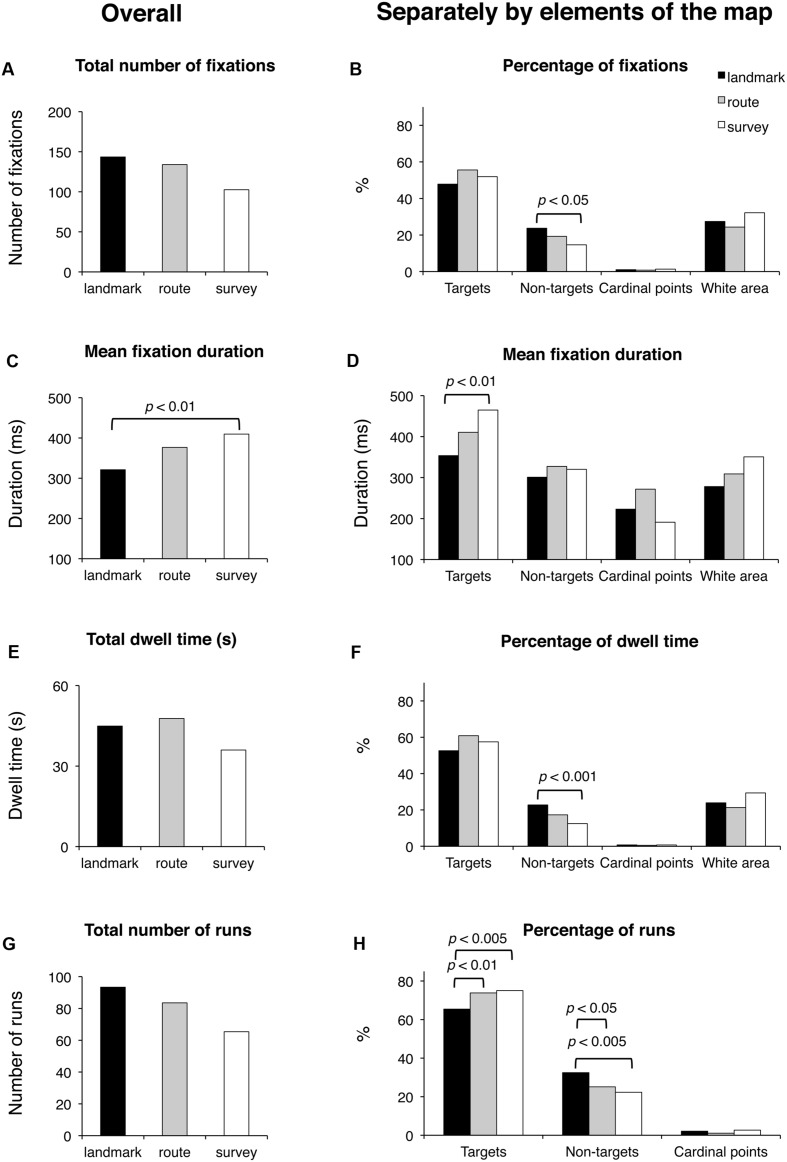
**Delayed recall phase.** Main results are shown for overall (left panels) and for distinct elements of the map (target and non-target blocks, cardinal points, and white area; right panels) for the following eye movement parameters: total number of fixations **(A)**, percentage of fixations **(B)**, mean fixation duration **(C,D)**, total dwell time **(E)**, percentage of dwell time **(F)**, total number of runs **(G)**, and percentage of runs **(H)**.

Results are reported in **Figure 6**. Groups were comparable for the total time of execution (*F*_(2_,_48)_ < 1; *p* = 0.59), the time elapsing from the first to the last selected block (*F*_(2,48)_ = 1.65; *p* = 0.20), and the total number of fixated targets (*F*_(2,48)_ < 1; *p* = 0.60). They tended to differ in the case of fixated non-targets (*F*_(2,48)_ = 2.73; *p* < 0.075). Concerning the selection of either the correct blocks (regardless of the sequence) or the distractors, the groups differed significantly in the number of both selected target (*F*_(2,48)_ = 8.86; *p* < 0.001) and non-target blocks (*F*_(2,48)_ = 4.50; *p* < 0.05); *post hoc* analyses showed that SS selected more correct targets (7.6) than LS (6.2; *p* < 0.005) and RS (6.9; *p* < 0.05); RS in turn differed from LS (*p* < 0.05); in the case of non-targets, LS (1.4) differed from SS (0.4; *p* < 0.01), but not from RS (0.9; *p* = 0.13), which was comparable to LS (*p* = 0.15).

The percentage of participants that were able to recall the map correctly (i.e., the target blocks in the correct sequence) was 5.0, 41.2, and 64.3%, for the LS, RS, and SS groups, respectively.

Groups did not differ significantly in the total number of fixations and the percentage of fixations on the targets (in both cases, *F*_(2_,_48)_ < 1; *p* = 0.60 and *p* = 0.11, respectively). There was a significant group difference for the percentage of fixations on the non-targets (*F*_(2,48)_ = 6.03; *p* < 0.005); *post hoc* showed that SS had a smaller percentage (14.6) with respect to LS (23.7; *p* < 0.005) and tended to differ significantly from RS (19.3; *p* = 0.08), while RS and SS were comparable (*p* = 0.10). The group differences for the cardinal points and the white area were not significant (*F*_(2,48)_ < 1 and *F*_(2,48)_ = 1.70; *p* = 0.62 and *p* = 0.19, respectively). Groups differed significantly in the average fixation duration (*F*_(2,48)_ = 4.67; *p* < 0.05); *post hoc* showed that LS had a shorter duration (321 ms) than SS (410 ms; *p* < 0.01), and tended to make shorter fixations than RS (377 ms; *p* < 0.068), who did not differ from SS (*p* = 0.27). Group difference was significant for the duration of fixations on targets (*F*_(2_,_48)_ = 4.25; *p* < 0.05); *post hoc* showed that LS had shorter durations (354 ms) than SS (465 ms; *p* < 0.01) but not RS (411 ms; *p* = 0.14), who in turn did not differ from SS (*p* = 0.16). The group difference for cardinal points (*F*_(2,48)_ = 2.86; *p* = 0.078) marginally approached significance. The group difference for white area (*F*_(2,48)_ = 3.02; *p* = 0.058) tended to significance. The groups had comparable total dwell times (*F*_(2,48)_ < 1; *p* = 0.68) and comparable percentages of dwell time for targets (*F*_(2,48)_ = 2.23; *p* = 0.12), cardinal points (*F*_(2,48)_ < 1; *p* = 0.78), and white area (*F*_(2,48)_ = 1.85; *p* = 0.18). They differed in the percentages of dwell time for non-targets (*F*_(2,48)_ = 6.64; *p* < 0.005); *post hoc* analysis showed that LS (22.8) had greater percentages than SS (12.5; *p* < 0.001) and tended to have greater percentages than RS (17.3; *p* = 0.058), who in turn marginally tended to differ from SS (*p* = 0.089). The groups were comparable for the total number of runs (*F*_(2,48)_ < 1; *p* = 0.58) but significantly differed for the percentage of runs for targets (*F*_(2,48)_ = 6.05; *p* < 0.005) and non-targets (*F*_(2,48)_ = 5.84; *p* < 0.01). *Post hoc* showed that LS made a smaller percentage of runs for targets (65.4) than RS (73.4; *p* < 0.01) and SS (75.0; *p* < 0.005), and a greater percentage of runs for non-targets (32.5) than RS (25.1; *p* < 0.05) and SS (22.3; *p* < 0.005), who were comparable for both kinds of element (*p* = 0.38). The groups did not show differences in the percentage of runs in the case the cardinal points (*F*_(2,48)_ = 1.16; *p* = 0.32). Finally, saccade amplitude was comparable in all three groups (*F* < 1; *p* = 47).

## Discussion

In the present study we tested the hypothesis that the different levels of navigational ability that characterize different types of SCSs could correspond to differences in the visual exploration of environmental information. More specifically, we hypothesized that SCSs affect the way an individual observes novel environmental features during the learning of an environment or a route, so that each SCS corresponds to a typical pattern of visual exploration. We expected that individuals characterized by a more efficient SCS, namely the SS, would show a more complex and wider pattern of visual exploration allowing the acquisition of the detailed knowledge necessary for the development of an allocentric cognitive map. In order to test this hypothesis, we asked individuals with different SCSs to learn and recall a path on a map during eye movement recording, a direct measure of the pattern of visual exploration. Data collected include the time spent on fixating salient and non-salient features of the environmental map, and the number of “comings back” to look at a given feature. Results show the presence of specific patterns of eye movements in the three different SCSs, supporting our hypothesis that SCS is reflected in the way individuals visually explore the environments.

As expected, accuracy in solving the delayed recall task differentiated across groups: only one LS participant (5%) was able to recall the map correctly. On the contrary, the RS and SS individuals were more proficient in solving the task, and more than 60% of the SS participants were able to reproduce the sequence correctly. These results could be also due to the different number of males and females in the three cognitive styles but, as reported by Nori and Giusberti (2003), Nori et al. (2006), and Nori and Piccardi (2011), when cognitive style is considered, gender differences disappeared in solving spatial problems. Therefore, we are inclined to believe that the non-homogeneous distribution of males and females within the three cognitive styles do not affect performance, since a LS male has a performance equivalent to that of a LS female, that is also true for a SS male and a SS female (see also Nori and Piccardi, 2011).

**FIGURE 7:**
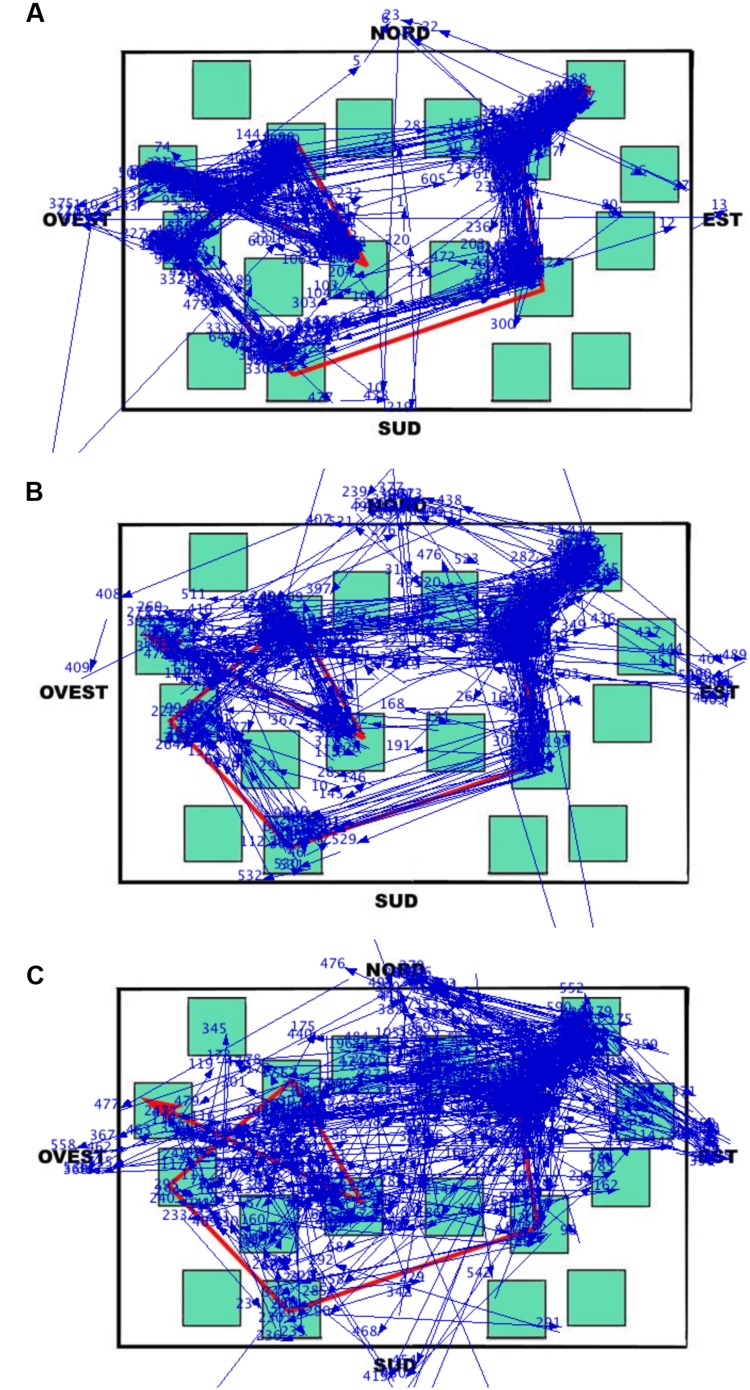
**Learning phase.** Results of Data Viewer analysis show the eye movement pattern for three representative participants: **(A)** Landmark; **(B)** Route; **(C)** Survey. Each segment represents a saccadic eye movement (the arrow indicates direction) connecting two successive fixations (not shown). Nord = North; Sud = South; Est = East; and Ovest = West.

During the learning phase, the overall trend (i.e., when the eye movements were analyzed without separating the different elements of the map) was that the LS group made the greatest number of fixations and runs, and had the shortest fixation durations; the groups were comparable in the case of dwell time (since the dwell time is the sum of all fixation durations, the number of fixations counterbalanced the fixation duration in each group). This trend differentiated when data were analyzed considering the targets, the non-targets, and the white area of the map separately (the cardinal points did not show any remarkable observation pattern). Peculiarly, the SS group spent more viewing time (i.e., the dwell time) than the other groups on the non-targets and the white area of the map, and less time than the other groups on targets. The dwell time on the non-targets and the white area was in fact greater for SS both in relative (the percentages reported in the analysis) and absolute terms (not reported). This group therefore observed these elements with more fixations and longer durations than the other groups (the dwell time being obtained by multiplying the number of fixations by the fixation duration). The overall result is that SS individuals studied the path by locating fixations in a more diffuse manner (see **Figure 7**), often including the non-targets and the white area (i.e., SS did not remain tied to the red path but they scanned the overall surface of the map; they looked at the white parts as these were as informative as the target sequence highlighted by the red line). This ‘spread’ looking behavior probably granted a better spatial representation of the whole map, thus determining the achievement of the best performance in this group. This eye movement pattern clearly differed from those of the other groups, especially from that of LS, who had the worst recall performance, produced the greatest number of fixations and shortest fixation durations during the learning phase, and was characterized by the highest number of runs, that is, showed numerous returns to single elements of all kinds. The RS group’s eye movement pattern was overall midway between that of LS and SS, being more similar to SS when considering overall parameters (i.e., without separating the different elements of the map) and more similar to LS when analyzing the different components of the map.

**FIGURE 8:**
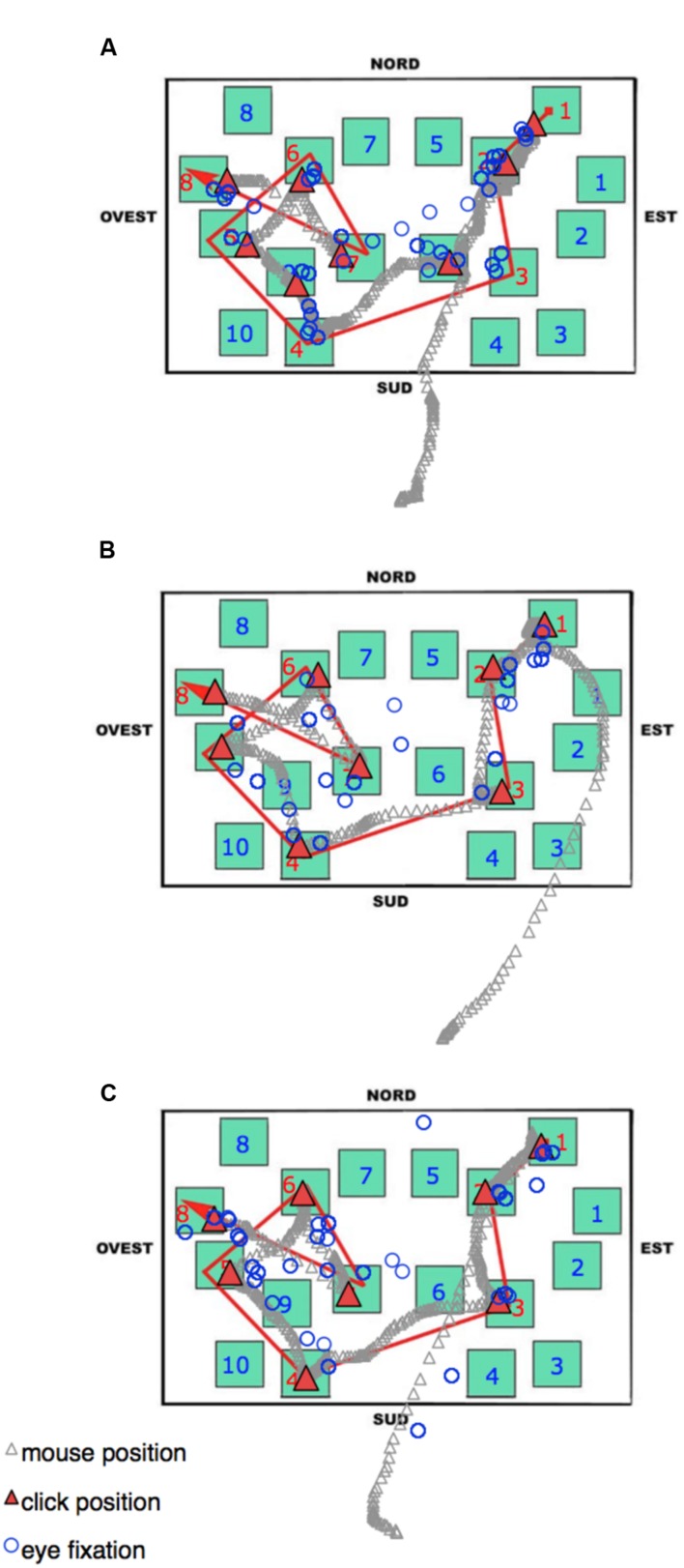
**Delayed recall phase.** An Excel graph based on Data Viewer results was used to visualize the behavioral and eye movement performance in the same three representative participants as **Figure 7**: **(A)** Landmark; **(B)** Route; **(C)** Survey. Open circles represent the pattern of fixations. The gray stream of triangles is the dislocation of the mouse during the task, and the red triangles highlight the mouse position when the participant pressed the mouse button to select a block. Nord = North; Sud = South; Est = East, and Ovest = West.

During the delayed recall, the total number of fixations, the dwell time, and the number of runs were comparable among all three groups. As in the learning phase, the LS group showed the shortest fixation durations, and this seems to be a characteristic that could be worth investigating in other kinds of task (see **Figure 8** to visualize behavioral and eye movement performance in the three groups). The time to execute the task (both overall and from the moment a participant started to select the blocks by clicking the mouse) was statistically comparable among the groups; nevertheless it is noteworthy that the SS group was faster (25–40%) than the other groups. The only other peculiarity observed during the delayed recall test is that LS showed a higher amount of time devoted to non-targets (with more fixations, runs, and dwell time) than the other groups. We interpreted this pattern as an index of hesitation: in fact, while on the one hand, SS individuals significantly made fewer fixations on the non-targets compared to RS and LS participants (probably meaning that they were able to quickly identify the target without checking the not pertinent blocks), on the other hand, LS needed to check the available adjacent alternatives (i.e., the non-targets) when attempting to reconstruct the path.

To synthesize, through eye movement recordings it was possible to detect significant differences among the three SCSs at the level of an objective and measurable physiological parameters. The main difference concerned the distribution of fixations during the learning phase: the SS group is characterized by a broader and more comprehensive explorative pattern; on the contrary, LS participants focused their exploration mainly on the path targets. Therefore, the present results confirm the presence of different cognitive styles and the different modalities adopted to analyze spatial information: LS acquire spatial information by fixing landmarks and their characteristics irrespective of their spatial position whereas SS analyze the environment considering the relationship among the elements and analyzing the environment as a whole.

Our data support the suggestion that differences in acquiring environmental information could be due to differences in paying attention to the various cues in the environment. Indeed, Denis et al. (1999) demonstrated that LS individuals, despite having a poor representation of spatial components, are able to move successfully in the environment. Generally speaking, LS have a good representation of landmarks, but are not sure how to reach them. They move in the environment using a sort of trial-and-error strategy. Indeed, we found that also when studying a map they prefer to focus their attention on landmarks regardless of their spatial position, differently from RS and SS. In line with Nori et al. (2006) SS individuals, even though more accurate than the other two groups, consider more information than RS and LS to build up their environmental representation, spending more time on expressing directional judgments from a point of view different from the one they had started with (Nori et al., 2006) and this is confirmed in the present study by the fact that they produce more fixation points on the map.

Our results add to those of neuroimaging in showing individual differences at the level of eye movements that have also been found at an anatomical level. Indeed some studies show gray and white matter differences or different brain activations in medial temporal lobe structures (MTL) between good navigators, who are usually individuals with good survey competences, and bad navigators, who are individuals with poor survey competences (Auger et al., 2012; Auger and Maguire, 2013; Arnold et al., 2014; Wegman et al., 2014; Sulpizio et al., 2016). For example, Auger et al. (2012) found reduced retrosplenial cortex activation in bad navigators compared to good navigators in an fMRI navigational task, while Wegman et al. (2014) observed trends toward higher gray matter volume in the right anterior parahippocampal gyrus and rhinal cortex for good versus bad navigators and in the right caudate nucleus for bad versus good navigators. In addition, Sulpizio et al. (2016) showed that the functional connectivity between the posterior hippocampus and the retrosplenial complex was higher in good than in poor navigators. It remains to be systematically addressed whether and how individual differences that we have showed here at the level of eye movements could be directly linked to such anatomical differences. We still know little about the relationship between MTL, which are involved in visuo-spatial memory and that show anatomical differences between good and bad navigators, and the oculomotor system, but recent studies point to an interaction between memory and looking behavior as well as between the underlying neural systems (see Meister and Buffalo, 2016 for a review). Indeed, the MLT activities reflect the eye position within the visual scene (Nowicka and Ringo, 2000; Killian et al., 2012), and looking behavior is in turn guided by memory and is sensitive to damage of MLT structures (Smith and Squire, 2008; Hannula et al., 2010). Specifically, eye movements can also be pre-planned independently by current eye position thanks to an allocentric visuo-spatial map of the environment built up in MLT structures which could inform looking (e.g., a non-retinal map could be used to direct eye movements to a target not currently in sight; see Meister and Buffalo, 2016). Crucial in this process could be the role of the posterior cingulate cortex and the retrosplenial cortex that seem to be involved in format transformation (i.e., egocentric-allocentric transformation; Burgess et al., 2001; Dean and Platt, 2006; Byrne et al., 2007). Differences in eye movement patterns according to the cognitive styles could reflect thus anatomical differences in these crucial areas, and this ties in well with the fact that good and bad navigators show anatomical differences in retrosplenial cortex. In addition following Meister and Buffalo (2016) areas involved in saccadic eye movements and in the oculomotor decision of when and where to look (e.g., prefrontal cortex, posterior parietal cortex, frontal eye fields, and superior colliculus) could all be possible targets of the MLT output that influences eye movements. Specifically, the so-called command neurons in parietal cortex could be targets for “the MLT output that influences the saccadic decision of *where* to look” while the MLT projection to locus coeruleus can influence “*whe*n to look” possibly causing a slowing in the rate of making saccades for remembered stimuli or changing in pupil size (Meister and Buffalo, 2016). Future research should aim to investigate possible anatomical differences between good and bad navigators in connectivity between MLT and oculomotor structures involved in “where and when” to look.

The present results also have great clinical relevance with respect to specific navigational learning disabilities such as DTD. People affected by DTD show navigational deficits in the context of normal intellectual ability and in the absence of any known perinatal, neurological, or psychiatric disorder. Since 2009 several cases of the DTD have been described (Iaria et al., 2009; Bianchini et al., 2010, 2014; Iaria and Barton, 2010; Palermo et al., 2014b,a; Kim et al., 2015; Nemmi et al., 2015).

At present, there is evidence that different kinds of DTD and different degrees of severity exist (Bianchini et al., 2014; Palermo et al., 2014b) and that this disorder is widespread among the population. Indeed, Iaria and Barton (2010), through a specific website developed for recruiting people with navigational difficulties, found 120 cases of people who fulfilled the criteria for a diagnosis of DTD. Both Palermo et al. (2014b) and Nemmi et al. (2015) used fMRI to investigate which brain areas were activated during a route-following task in two different individuals with DTD (Mr. L.A. and Dr. W.A.I., respectively). Palermo et al. (2014b) found that Mr. L.A. showed activation in the occipital areas, involved in low-level perceptual analysis of the stimuli, but showed no activation in the areas activated in controls with regard to route knowledge. In line with this result, also Dr. W.A.I. showed brain activations in the occipital lobe (i.e., the calcarine and lingual gyri), regions probably related to the first level of the landmark identification process, and showed no activation of the medial temporal areas known to be involved in navigational processes (i.e., landmark recognition, and landmark-based navigation). Instead, to the best of our knowledge, only one study has described the pattern of eye movements in exploring familiar and unfamiliar landmarks in a child with DTD (Piccardi et al., under review), but no studies have analyzed eye movements in individuals with DTD during path learning. Although our knowledge of this aspect is limited, it is feasible to suggest that individuals with DTD, like individuals affected by other selective developmental deficits such as developmental prosopagnosia (Duchaine, 2000; Behrmann and Avidan, 2005; Pizzamiglio et al., 2015), may show a peculiar pattern of eye movements. Specifically, individuals with developmental prosopagnosia when exploring a face show a large percentage of fixations on external features, and they are particularly poor at exploring the eye region, that is a crucial area in recognizing familiar faces (e.g., Schmalzl et al., 2008). Concerning DTD, we characterized this disorder with a greater number of fixations and longer fixation durations (Piccardi et al., under review), while longer fixation durations and the fixation distribution restricted in the lower parts of the face, but not the number of fixations, characterized the eye movement pattern in the prosopagnosia case study (Pizzamiglio et al., 2015). At a deeper level of analysis, current findings on the differences in the visual exploration of a map in healthy individuals without DTD, but with different levels of proficiency in navigation, strongly suggest that the first visual analysis of navigational stimuli should be systematically investigated in DTD and that, as in healthy individuals with different cognitive spatial styles, individuals with different types of DTD could be characterized by different pathological patterns of eye movements. In the near future eye movement recordings could be an important tool in support of the neuropsychological testing for increasing our knowledge and defining the subtype of DTD, a modality surely less expensive than fMRI and easier to use at all ages.

## Author Contributions

Laura Piccardi: conceived, designed, and performed the experiment, wrote the paper; ML: conceived, designed, performed, and analyzed eye movements recording and wrote the part of the eye movements; RN: conceived, designed, and wrote the paper; Liana Palermo: conceived, designed, and wrote the paper; FI: collected all data on cognitive styles and analyzed them; CG: conceived, designed, and wrote the paper.

## Conflict of Interest Statement

The authors declare that the research was conducted in the absence of any commercial or financial relationships that could be construed as a potential conflict of interest.
